# 17‐ *epi* ‐Melianodiol, a new protolimonoid from *Melia azedarach* fruits, exhibits larvicidal activity and is associated with oxidative imbalance and midgut epithelial damage in *Aedes aegypti* larvae

**DOI:** 10.1002/ps.70968

**Published:** 2026-05-27

**Authors:** Kethleen Duarte Crespo Soares, Thalya Soares Ribeiro Nogueira, Maria Aparecida Aride Bertonceli, Renata Rodrigues da Silva Robaina, Leticia Oliveira da Rocha, Francisco José Alves Lemos, Kátia Valevski Sales Fernandes, Raimundo Braz‐Filho, Ivo José Curcino Vieira

**Affiliations:** ^1^ Universidade Estadual do Norte Fluminense Darcy Ribeiro Setor de Química dos Produtos Naturais, Laboratório de Química Bio‐orgânica Campos dos Goytacazes Brazil; ^2^ Universidade Estadual do Norte Fluminense Darcy Ribeiro Laboratório de Química e Função de Proteínas e Peptídeos, Centro de Biociências e Biotecnologia Campos dos Goytacazes Brazil; ^3^ Universidade Estadual do Norte Fluminense Darcy Ribeiro Laboratório de Biologia Celular e Tecidual, Centro de Biociências e Biotecnologia Campos dos Goytacazes Brazil; ^4^ Universidade Estadual do Norte Fluminense Darcy Ribeiro Laboratório de Biotecnologia, Centro de Biociências e Biotecnologia Campos dos Goytacazes Brazil; ^5^ Departamento de Química, Instituto de Química Universidade Federal Rural do Rio de Janeiro Seropédica Brazil

**Keywords:** *Aedes aegypti*, 17‐*epi*‐melianodiol, larvicidal activity, *Melia azedarach*, midgut epithelial damage, oxidative imbalance

## Abstract

**BACKGROUND:**

In Brazil, *Aedes aegypti* is the primary vector of arboviruses such as dengue, chikungunya, yellow fever, and Zika virus, posing a major public health concern, particularly in underdeveloped regions where insecticide resistance and the circulation of highly virulent strains are increasing. In search of alternative and sustainable control strategies, this study evaluated the biolarvicidal potential of compounds from the fruits of *Melia azedarach* against third‐instar *Ae. aegypti* larvae, focusing on larvicidal efficacy and associated physiological alterations.

**RESULTS:**

In this study, the crude methanolic extract caused 100% larval mortality at 100 ppm. After liquid–liquid partitioning, the dichloromethane fraction exhibited the highest larvicidal activity (86.0% mortality). Bioassay‐guided fractionation led to the isolation of the new compound 17‐*epi*‐melianodiol (1), a protolimonoid, which showed relevant larvicidal activity (LC₅₀ = 41.5 ppm; LC₉₀ = 64.7 ppm). Larvae exposed to 1 displayed pronounced morphological alterations, including cuticular damage and extensive vacuolization, as revealed by optical and transmission electron microscopy. In addition, 1 significantly increased reactive oxygen species (ROS) levels in the larval midgut, indicating the occurrence of oxidative imbalance associated with tissue damage.

**CONCLUSIONS:**

In summary, these combined results suggest that 17‐*epi*‐melianodiol is a novel plant‐derived larvicide with promising potential, contributing to our understanding of its physiological effects on *Ae. aegypti* larvae. © 2026 The Author(s). *Pest Management Science* published by John Wiley & Sons Ltd on behalf of Society of Chemical Industry.

## Graphical abstract

The bio‐guided phytochemical study resulted in the new compound 17‐*epi*‐melianodiol. The compound eliminated 100% of *Aedes aegypti* larvae at 100 ppm, causing pronounced morphological changes, cuticular damage, and extensive vacuolization.

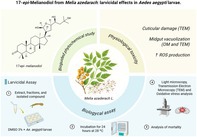

## 1 INTRODUCTION

*Aedes aegypti* is an anthropophilic mosquito involved in the transmission of several arboviruses, including dengue, chikungunya, Zika virus, and yellow fever.^1^ The persistent increase in arboviral outbreaks in tropical countries has reinforced the need for effective and sustainable vector control strategies. According to the epidemiological bulletin issued by the Brazilian Ministry of Health, an exponential increase has been observed over the years in the number of arboviral cases in the country. This trend has resulted in recurring epidemics and significant impacts on mortality rates.^1^

Data from the World Health Organization (WHO) indicate that, from 2025, approximately 1.6 million probable cases of dengue were reported across Brazil. Among these, 1793 resulted in deaths^1^ Chikungunya and Zika viruses together accounted for approximately 129 000 probable cases and 121 deaths. During the same period, yellow fever recorded 120 confirmed cases and 48 deaths, corresponding to a case fatality rate of nearly 40%.^1^

Currently, the control of arbovirus transmission involves mechanical, biological, environmental, chemical, and genetic methods.^2^ Two dengue vaccines have been licensed by the World Health Organization (WHO): Dengvaxia (CYD‐TDV), developed by Sanofi Pasteur, and Qdenga (TAK‐003), developed by Takeda. However, the use of CYD‐TDV requires pre‐vaccination screening and is restricted to specific age groups, limiting its broad implementation. Similarly, TAK‐003 is recommended primarily for children and adolescents in regions with high dengue transmission and is not currently indicated for younger children or older adults. Although vaccines represent an important advancement in dengue prevention, vector management remains the primary strategy for controlling dengue and other urban arboviruses, including chikungunya and Zika, particularly in endemic regions.^3^ The main approach targets the immature mosquito stages through the use of larvicides, such as organochlorines, organophosphates, carbamates, and synthetic pyrethroids.^2^ However, conventional larvicides have shown decreasing effectiveness due to the growing incidence of insecticide resistance, toxicity to non‐target organisms, and environmental contamination caused by persistent degradation products.^2^, ^4^, ^5^ These challenges highlight the urgent need to identify new larvicides with reduced toxicity and greater environmental safety. In this context, plant‐derived bioactive compounds have emerged as environmentally compatible alternatives for mosquito control.^4^, ^5^

According to Araújo and collaborators^6^ several factors support the use of medicinal plants in the health field. The Meliaceae family, widely recognized as a rich source of structurally diverse bioactive compounds, predominates in tropical and subtropical regions and comprises approximately 50 genera and about 1400 species.^7^ In Brazil, this family is particularly well represented in the Amazon and Atlantic Forest phytogeographic domains.^8^

*Melia azedarach* (Meliaceae) is a tropical species native to Asia that has spread across various tropical and subtropical regions of Africa, the Americas, and Australia.^9^ It is known for its natural resistance to pests and diseases and for its production of a wide range of secondary metabolites, including steroids, triterpenes, flavonoids, and limonoids.^6^, ^10^ Although limonoids and protolimonoids have been widely reported as insecticidal, such as meliacarpins, trichilinins, azedarachins, melianodiol, meliantriol, etc.^11^, ^12^

Larvicidal activity of *M. azedarach* extracts against mosquito larvae have been reported in the literature;^9^, ^13^ however, the larvicidal activity of individual constituents from *M. azedarach* fruits remains insufficiently explored. In this context, the present study integrates phytochemical investigation and bioassay‐guided fractionation to identify active constituents from the methanolic fruit extract and to evaluate their effects on third‐instar *Ae. aegypti* larvae.

## 2 MATERIALS AND METHODS

### 2.1 Biological materials

#### 2.1.1 *Melia azedarach* L.

Fruits of *M. azedarach* (SisGen code A82E45C and ICMBio code 102642‐1) were collected in March 2022 in Campos dos Goytacazes, Rio de Janeiro, Brazil (latitude 21°75′20.62″ S, longitude 41°34′63.34″ W). The fruits were dried at 28 °C and stored until further analysis. A voucher specimen (HUENF 15745) is deposited in the Herbarium of the Universidade Estadual do Norte Fluminense Darcy Ribeiro (UENF).

#### 2.1.2 Aedes aegypti

Larvae of *Ae. aegypti* Rockefeller strain were obtained from the insectarium maintained at the Biotechnology Laboratory (LBT) of the State University of Northern Rio de Janeiro Darcy Ribeiro (UENF). Adult mosquitoes were reared at room temperature (27 ± 3 °C) and supplied with a sterile 10% sucrose solution in an environment containing sterile distilled water to maintain adequate humidity. Larvae were fed sterile, finely ground commercial fish food. Pupae were washed and transferred to sterile distilled water and kept separately until adult emergence. Oviposition was induced by offering mouse or lamb blood meals. Three to 4 days after feeding, females deposited eggs on moist filter paper strips. Two days after oviposition, the dried eggs were transferred to plastic containers containing deoxygenated distilled water to promote larval hatching.^14^ The establishment and maintenance of the mosquito colony were approved by the Animal Ethics Committee of UENF (CEUA/CBB/UENF, approval no. 011/2023).

### 2.2 General phytochemical procedures

NMR spectra were acquired by using Bruker Ascend 500 (500 MHz for ^1^H and 125 MHz for ^13^C), using chloroform (CDCl_3_) as solvent containing TMS (tetramethylsilane) as an internal standard. Chemical shifts (*δ*) in ppm and coupling constants (*J*) in Hz. HR‐ESI‐MS data were acquired using an micrOTOF‐Q II Bruker Daltonics mass spectrometer, with the use of the positive ion mode of analysis. Column chromatography (CC) was performed on silica gel 60 (0.063–0.200 mm, MERCK). TLC plates contained with silica gel 60 F_254_ (MERCK) were used for analytical analysis. Analytical grade methanol (99.8%), ethyl acetate (99.5%), dichloromethane (99.5%), acetone (99.5%), *n*‐hexane (98.5%) and *n*‐butanol (99.4%) used for extraction and isolation were purchased from Synth (São Paulo, Brazil).

### 2.3 Extraction and isolation

The dried fruits of *M. azedarach* (1.0 kg) were powdered and extracted three times with methanol at room temperature. The methanolic extract was concentrated under reduced pressure to yield the crude extract (280.0 g). A portion of the methanolic extract (40.0 g) was suspended in distilled water and successively partitioned with CH_2_Cl_2_, EtOAc, and *n*‐BuOH. The dichloromethane partition (PD ‐16.63 g) was subjected to silica gel column chromatography (CC) using a polarity gradient of CH_2_Cl_2_–MeOH (from 100:0 to 0:100, v/v), affording nine fractions. Fraction six (PD6–0.940 g) was further chromatographed on silica gel, eluted with a CH_2_Cl_2_–MeOH gradient (100:0 to 0:100, v/v), to yield ten subfractions. Subfraction PD6.3 (0.168 g) was subsequently fractionated on silica gel using a hexane–EtOAc gradient, resulting in the compound isolated as protolimonoid 17‐*epi*‐melianodiol (PD6.3.4–0.028 g).

### 2.4 Chemical characterization

#### 2.4.1 ^1^H NMR (CDCl_3_ , 500 MHz)

*δ*_H_ 2.01 (*m*; Ha‐1), *δ*_H_ 1.49 (*m*; Hb‐1), *δ*_H_ 2.77 (*dd* = 14.6 and 5.5 Hz; Ha‐2), *δ*_H_ 2.26 (*dt* = 14.6 and 3.5; Hb‐2), *δ*_H_ 1.74 (*t* = 7.6 Hz; H‐5), *δ*_H_ 2.15 (*m*; Ha‐6), *δ*_H_ 2.05 (*m*; Hb‐6), *δ*_H_ 5.34 (*m*; H‐7), *δ*_H_ 2.31 (*m*; H‐9), *δ*_H_ 1.60 (*m*; 2H‐11), *δ*_H_ 1.75 (*m*; Ha‐12), *δ*_H_ 1.45 (*m*; Hb‐12), *δ*_H_ 1.55 (*m*; 2H‐15), *δ*_H_ 2.00 (*m*; Ha‐16), *δ*_H_ 1.90 (*m*; Hb‐16), *δ*_H_ 2.05 (*s*; H‐17), *δ*_H_ 0.86 (*s*; H‐18); *δ*_H_ 1.03 (*s*; H‐19), *δ*_H_ 2.01 (*s*; H‐20), *δ*_H_ 5.22 (*d* = 2.2 Hz; H‐21), *δ*_H_ 2.02 (*m*; Ha‐22), *δ*_H_ 1.92 (*m*; Hb‐22), *δ*_H_ 4.49 (*t* = 7.8 Hz; H‐23), *δ*_H_ 3.19 (*s*; H‐24), *δ*_H_ 1.30 (*s*; H‐26); *δ*_H_ 1.28 (*s*; H‐27); *δ*_H_ 1.05 (*s*; H‐28); *δ*_H_ 1.13 (*s*; H‐29) and *δ*_H_ 1.06 (*s*; H‐30). ^13^C NMR (CDCl_3_, 125 MHz): *δ*_C_ 38.52 (C‐1), *δ*_C_ 34.24 (C‐2), *δ*_C_ 216.49 (C‐3), *δ*_C_ 47.86 (C‐4), *δ*_C_ 52.47 (C‐5), *δ*_C_ 24.77 (C‐6), *δ*_C_ 118.18 (C‐7), *δ*_C_ 145.67 (C‐8), *δ*_C_ 48.40 (C‐9), *δ*_C_ 35.11 (C‐10), *δ*_C_ 17.77 (C‐11), *δ*_C_ 31,50 (C‐12), *δ*_C_ 43.55 (C‐13), *δ*_C_ 50.09 (C‐14), *δ*_C_ 34.88 (C‐15), *δ*_C_ 27.24 (C‐16), *δ*_C_ 45.36 (C‐17), *δ*_C_ 23.22 (C‐18), *δ*_C_ 12.73 (C‐19), *δ*_C_ 46.28 (C‐20), *δ*_C_ 97.69 (C‐21), *δ*_C_ 30.32 (C‐22), *δ*_C_ 78.70 (C‐23), *δ*_C_ 75.32 (C‐24), *δ*_C_ 73.37 (C‐25), *δ*_C_ 26.61 (C‐26), *δ*_C_ 26.69 (C‐27), *δ*_C_ 24.53 (C‐28), *δ*_C_ 21.57 (C‐29) and *δ*_C_ 27.46 (C‐30). The HR‐ESI‐MS/MS (positive mode) of *m*/*z* 511.3369 [M + Na]^+^ (calc. for C_30_H_48_O_5_Na 511.3394, Δ_*m/z*_ = −4.8).

### 2.5 Larvicidal effects induced by *M. azedarach* fruit extracts and the isolated pure compound

#### 2.5.1 Larvicide activity assay

Crude methanolic extract, its partitioned extracts (EtOAc, n‐BuOH, CH_2_Cl_2_, and H_2_O), chromatographic fractions of the bioactive extract, and the isolated pure compound were assayed for their mosquito‐larvicidal activity as per the WHO recommended procedures.^15^ A 100 ppm solution of the crude methanolic extract, liquid–liquid partitions, and chromatographic fractions were prepared in ultrapure water containing 3% DMSO and tested against ten third‐instar larvae of *Ae. aegypti*, and mortality observed after 24 h. Each assay was performed in triplicate, resulting in a total of 30 larvae per treatment, using 20 mL of solution per replicate.

For the liquid–liquid partitions and chromatographic fractions, 100 ppm solutions were similarly prepared. For the isolated compound, an initial stock solution (10 mg mL^−1^ in H_2_O containing 3% DMSO) was diluted with ultrapure water to obtain concentrations of 12.5, 25.0, 50.0, 75.0, and 100 ppm. In these assays, five third‐instar larvae were placed in each concentration, and all tests were performed in triplicate, totaling 15 larvae per treatment in 10 mL of solution per replicate.

The negative control consisted of ultrapure water containing 3% DMSO, while the positive control was a 100 ppm solution of the commercial larvicide spinosad (NATULAR™ DT). The larvae were exposed to the test solutions for 24 h at 28 °C. After incubation, mortality was assessed based on larval movement and confirmed under a stereomicroscope (ZEISS, Germany). Mortality data were statistically analyzed and mortality proportion was determined using Probit regression in RStudio with the *glm*() function (binomial distribution, probit link). Lethal concentration values (LC₅₀ and LC₉₀) were estimated from the parameters of the fitted model.

#### 2.5.2 Light microscopy and transmission electron microscopy (TEM)

For analysis by light microscope, ten larvae per treatment were dissected in 2% paraformaldehyde fixative solution and stored for 12 h at 4 °C. Then, the samples were dehydrated in a graded ethanol series (30–100%) and embedded in historesin (JB4 Poly‐sciences). Tissue sections of 5 μm thickness were stained with hematoxylin and eosin, then examined and photographed using a light microscope (Olympus BX60). For ultrastructural analyses of *Ae. aegypti*, larvae were dissected on 0.1 M sodium cacodylate buffer, pH 7.1, for 48 h, fixed in 2.5% glutaraldehyde and 4% paraformaldehyde. The samples were then washed three times in sodium cacodylate buffer, post‐fixed in 1% osmium tetroxide solution for 1 h and rinsed again with the same buffer. The material was dehydrated in an increasing acetone series (30, 40, 50, 70, 90 and 100%, for 1 h each) and embedded in Spurr resin and polymerized at 60 °C. Ultra‐thin sections of 70 nm were obtained and collected in 400 mesh copper grids and counterstained with 1% uranyl acetate and 5% lead citrate. The samples were then analyzed with a transmission electronic microscope at 60 kV (JEOL 1400 Plus). The control group and treated third instar larvae were compared.

#### 2.5.3 Oxidative stress analysis

For oxidative stress analysis, one test group and one negative control group, each containing six third instar *Ae. aegypti* larvae, were used. Initially, the groups were uniformly treated overnight with a solution containing 10 mM DTT,^16^ 0.03% agar and 50 μg mL^−1^ gentamicin. Subsequently, the larvae were rinsed with ultrapure water. The experimental group was exposed to the isolated compound at a concentration of 50 ppm, while the negative control group received only ultrapure water, for 24 h. Following this period, the ROS marker 2′,7′‐dichlorofluorescein diacetate (10 mM, Sigma Aldrich – D6883) was added for 2 h. Finally, the larvae were washed and transferred to microplate wells containing water. The fluorescence labeling was observed under a microscope (Axioplan, Carl Zeiss) and quantified using Zen 2.3 software (Blue Edition).

### 2.6 Statistical analysis

Variance analysis was conducted using one‐way ANOVA, followed by Tukey's post‐hoc test. Mortality data and mortality proportion were evaluated through Probit regression in RStudio, employing the *glm()* function with a binomial family and probit link.

## 3 RESULTS AND DISCUSSION

### 3.1 Bioassay‐guided isolation of larvicidal compound from *M. azedarach* fruits

**Figure 1:**
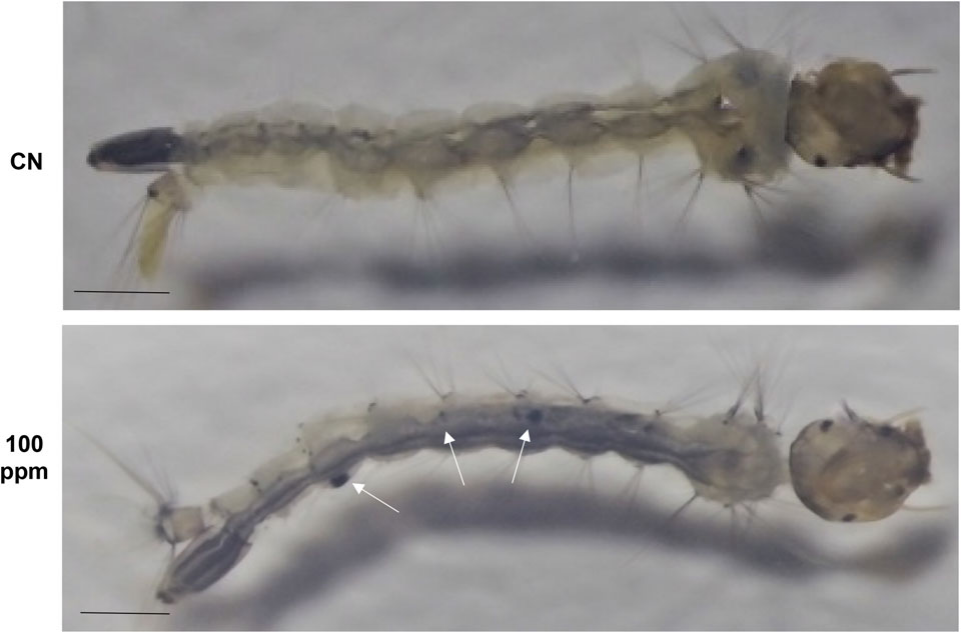
General morphological characteristics of third‐instar *Ae. aegypti* larvae exposed to 100 ppm of the methanolic extract from *M. azedarach* fruits, observed under a stereomicroscope. CN: Negative control DMSO‐3%. Scale bar = 1 mm.

**Figure 2:**
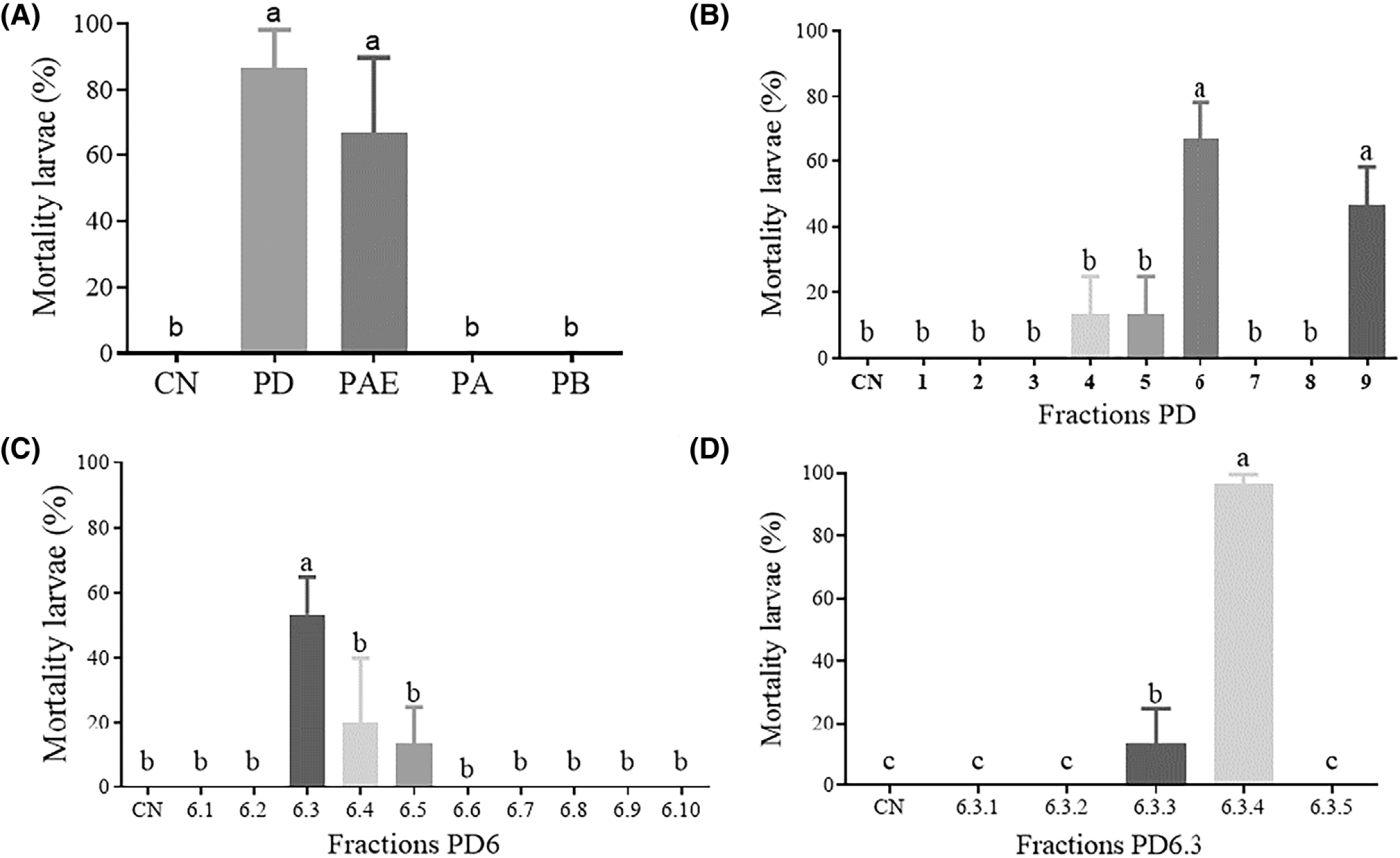
Larvicidal activity and bioassay‐guided fractionation of *M. azedarach* fruit extract against 3rd instar larvae of *Ae. aegypti*. (A) Larvicidal activity of crude extract partitions: PD (dichloromethane), PAE (ethyl acetate), PA (water) and PB (butanol), tested at 100 ppm for 24 h. CN: negative control (3% DMSO). (B) Larvicidal activity of fractions obtained from PD (fractions 1–9) tested at 100 ppm for 24 h. CN: negative control (3% DMSO). (C) Larvicidal activity of subfractions from PD6 (6.1–6.10) tested at 100 ppm for 24 h. CN: negative control (3% DMSO). (D) Larvicidal activity of subfractions from PD6.3 (6.3.1–6.3.5) tested at 100 ppm for 24 h. CN: negative control (3% DMSO). Each concentration was tested using five larvae per replicate in triplicate (n = 15 larvae per treatment). Different letters above the bars indicate statistically significant differences among treatments according to one‐way ANOVA (Tukey's multiple comparison test) (*P* < 0.0001).

The larvicidal assay using the crude methanolic extract of *M. azedarach* fruits at 100 ppm resulted in 100% larval mortality compared with the negative control (3% DMSO in ultrapure water). Morphological analysis of tested larvae under stereomicroscopy (Fig. 1) revealed the presence of darkened regions distributed along the larval body (white arrows), suggesting alterations in cuticle integrity. Similar patterns of cuticular darkening have been previously reported in larvae exposed to plant‐derived bioactive compounds.^14^ Larvicidal assays performed with the liquid–liquid partitions of the methanolic extract of *M. azedarach* fruits at 100 ppm identified the dichloromethane partition as the most toxic, causing 86.0% larval mortality, followed by the ethyl acetate partition with 66.6% lethality. In contrast, the aqueous and *n*‐butanol partitions exhibited no lethality compared to the negative control (3% DMSO) (Fig. 2(A)).

The dichloromethane partition (PD), which exhibited the highest larval mortality rate (86.0%,) was selected for further fractionation on a silica gel chromatographic column as the stationary phase. The process yielded nine subfractions, which were again bioassayed at 100 ppm against third‐instar *Ae. aegypti* larvae. Among these, fraction PD6 induced 66.6% larval mortality and was subjected to an additional chromatographic fractionation step (Fig. 2(B)). This subsequent process generated 10 subfractions, all tested at 100 ppm, with PD6.3 exhibiting the highest lethality (60.0%) (Fig. 2(C)). Further fractionation of fraction PD6.3 produced five subfractions, among which subfraction PD6.3.4 displayed a single‐compound chromatographic profile and caused 100% larval mortality when tested at an initial concentration of 100 ppm (Fig. 2(D)). This active subfraction was therefore selected for structural elucidation.

### 3.2 Structural characterization of compound 1

Based on the bioassay‐guided approach described above, subfraction PD6.3.4 yielded compound **1**. Spectroscopic analyses, including ^1^H and ^13^C NMR (1D and 2D) and HR‐ESI‐MS, allowed structural characterization, and the data were compared with previously reported melianodiol derivatives.^17^, ^18^ Complete spectral data are provided in the Supplementary Material (Table S1 and Figs S1–S7).

**Figure 3:**
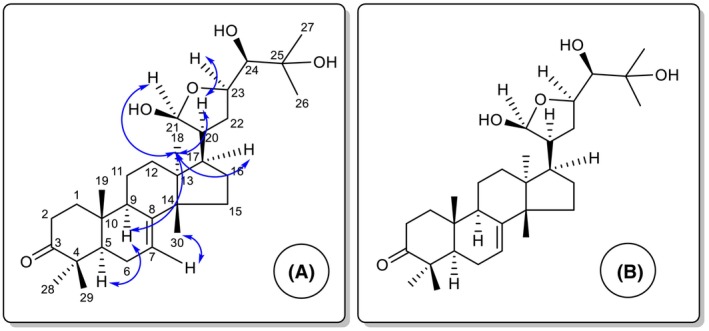
^1^H—^1^H— NOESY correlations observed to compound **1** (A) and the chemical structure of compound **1** (B).

**Figure 4:**
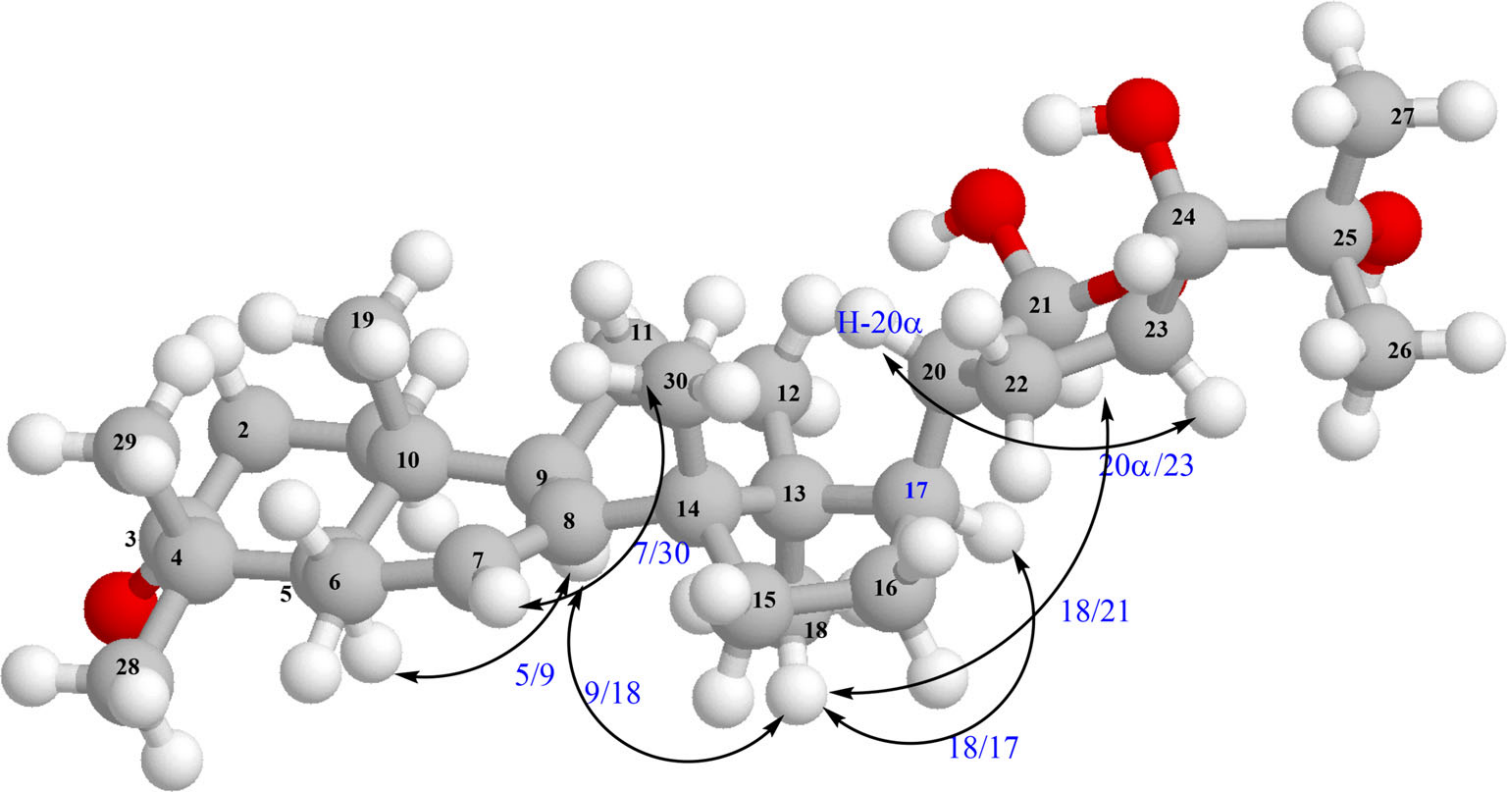
3D representation of the ^1^H—^1^H— NOESY correlations observed to compound **1**.

Previous reports describe the CH‐17–CH‐20 bond in melianodiol as having an *α* configuration.^18^ However, the ^1^H—^1^H NOESY spectrum of compound **1** revealed spatial interactions between H‐17 with 3H‐18 and H‐20; 3H‐18 with H‐20 and H‐23, consistent with a *β* orientation at CH‐17. These correlations (Figs 3 and 4) support the identification of compound **1** as 17‐*epi*‐melianodiol, representing a stereoisomer not previously described for this species.

### 3.3 Larvicidal activity and morphological alterations induced by compound 1

**Figure 5:**
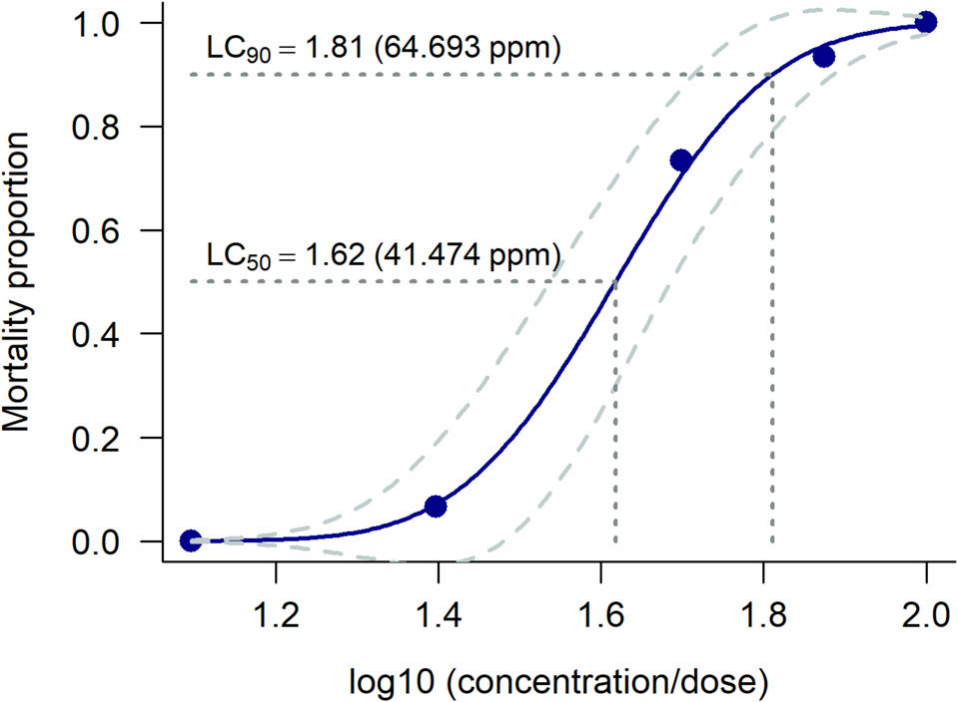
Larvicidal activity of isolated compound **1** against 3rd instar larvae of *Ae. aegypti*. All concentration (12.5, 25.0, 50.0, 75.0 and 100 ppm) were tested for 24 h. CN – Negative control DMSO‐3%. LC_50_ = 41.474 ppm and LC_90_ = 64.693 ppm, calculated using probit analysis performed in RStudio.

Compound **1** was evaluated for its larvicidal activity against third‐instar *Ae. aegypti* larvae at concentrations of 12.5, 25, 50, 75, and 100 ppm. A clear dose‐dependent increase in mortality was observed, with LC₅₀ and LC₉₀ values of 41.5 and 64.7 ppm, respectively (Fig. 5). Notably, this is the first report of the larvicidal activity of **1** against *Ae. aegypti* larvae and its associated morphological and physiological effects.

Under the same experimental conditions, the positive control (100 ppm) caused complete larval mortality and was used as a reference control. Compound **1** also achieved 100% mortality at 100 ppm and showed dose‐dependent activity (LC₅₀ = 41.5 ppm; LC₉₀ = 64.7 ppm). Although direct potency comparison was not the focus of this study, the distinct morphological and oxidative alterations observed suggest a physiological response different from the well‐established neurotoxic action of the commercial larvicide,^19^ supporting its potential relevance in complementary mosquito control strategies.^20^

Melianodiol (**2**) and melianotriol (**3**) were identified in *Guarea kunthiana* (Meliaceae) through bioactivity‐guided screening of 36 plant extracts. Although both compounds were obtained from an active fraction, compound **2** exhibited higher larvicidal activity than **3**.^12^ This difference may be related to structural variations, particularly the presence of a carbonyl group at C‐3 in ring A.^12^

**Figure 6:**
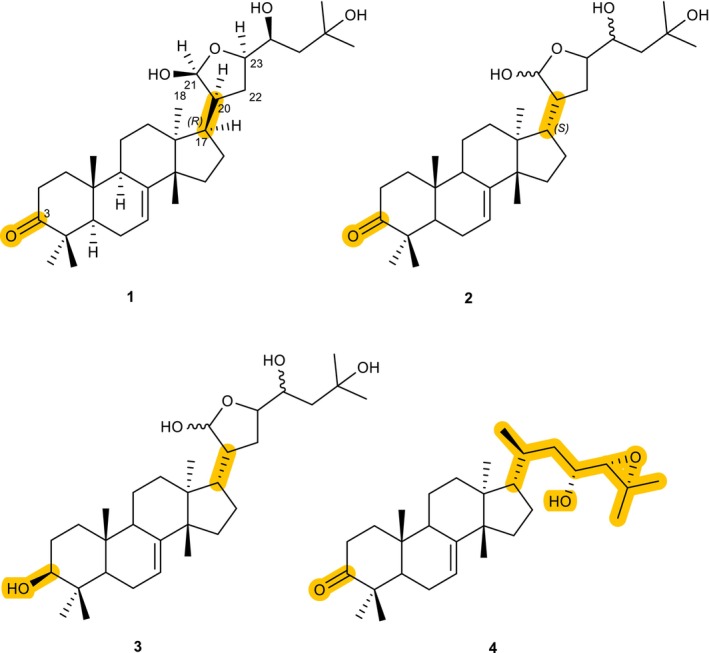
Chemical structures of 17‐*epi*‐melianodiol (**1**) melianodiol (**2**), melianotriol (**3**) and nilocetin (**4**). The stereochemical bonds of compound **1** are presented as defined in the present structural elucidation, whereas compound **2** and **3** retains the structure previously reported by Sarmento *et al*., (2016).

Nilocitin (**4**) is another protolimonoid reported as a potent larvicide against *Ae. aegypti*, showing higher activity than compound **1**.^21^ Comparative analysis of their chemical structures suggests that modifications in the side chain may significantly influence larvicidal potency. The structures of compounds **2**, **3**, and **4**, and their structural differences relative to **1**, are presented in Fig. 6.

The differences in biological activity between compound **1** and the melianodiol^12^ may also be associated with stereochemical variation at the CH‐17 – CH‐20 bond. In the present study, compound **1** is characterized by a 17*β* configuration (R enantiomer), whereas^12^ reported the 17*α* configuration (*S* enantiomer). As highlighted by Klebe,^22^ ‘the biological activities of enantiomers and diastereomers can vary greatly in both strength and quality.’ This stereochemical distinction, illustrated in Fig. 6, is supported by the NOESY correlations described in Section 3.2.

**Figure 7:**
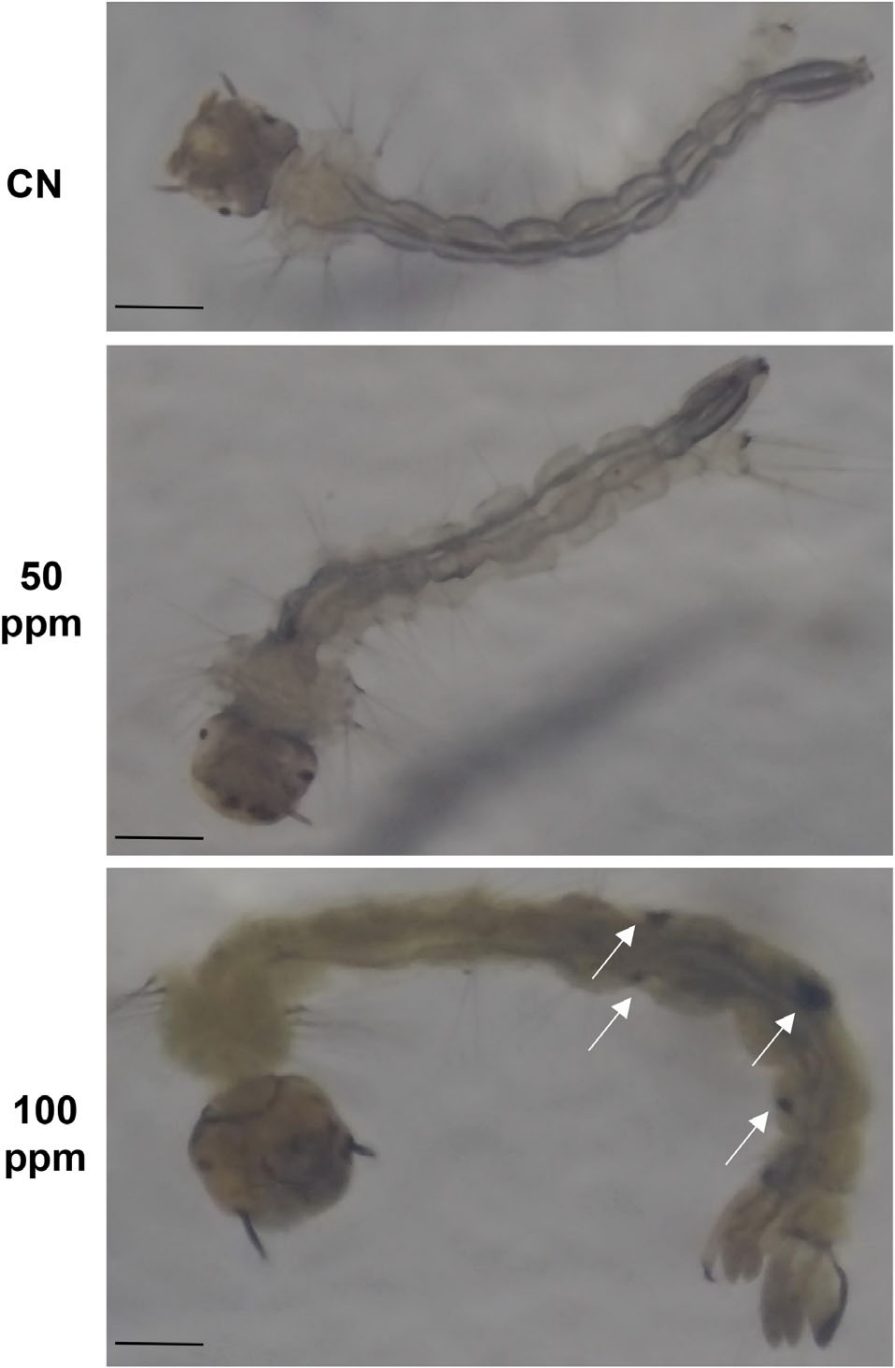
General morphological characteristics of third‐instar *Ae. aegypti* larvae exposed to 50 and 100 ppm of the isolated compound **1** from *M. azedarach* fruits, observed under a stereomicroscope. CN‐Negative control 3% DMSO. Scale bar = 1 mm.

Compound **1** is a tetracyclic triterpene, a class widely recognized for diverse biological activities, including insecticidal properties.^23^ Microscopic examination of larvae treated with 100 ppm of **1** (Fig. 7) revealed marked structural alterations in the cuticle, including loss of integrity, irregular thickening, and darkened regions along the body surface, particularly near the posterior midgut. Similar alterations were observed in larvae exposed to the methanolic extract (Fig. 1), supporting the interpretation that compound **1** substantially contributes to the larvicidal activity of the crude extract. These structural changes may compromise exoskeletal integrity and interfere with essential physiological processes.

**Figure 8:**
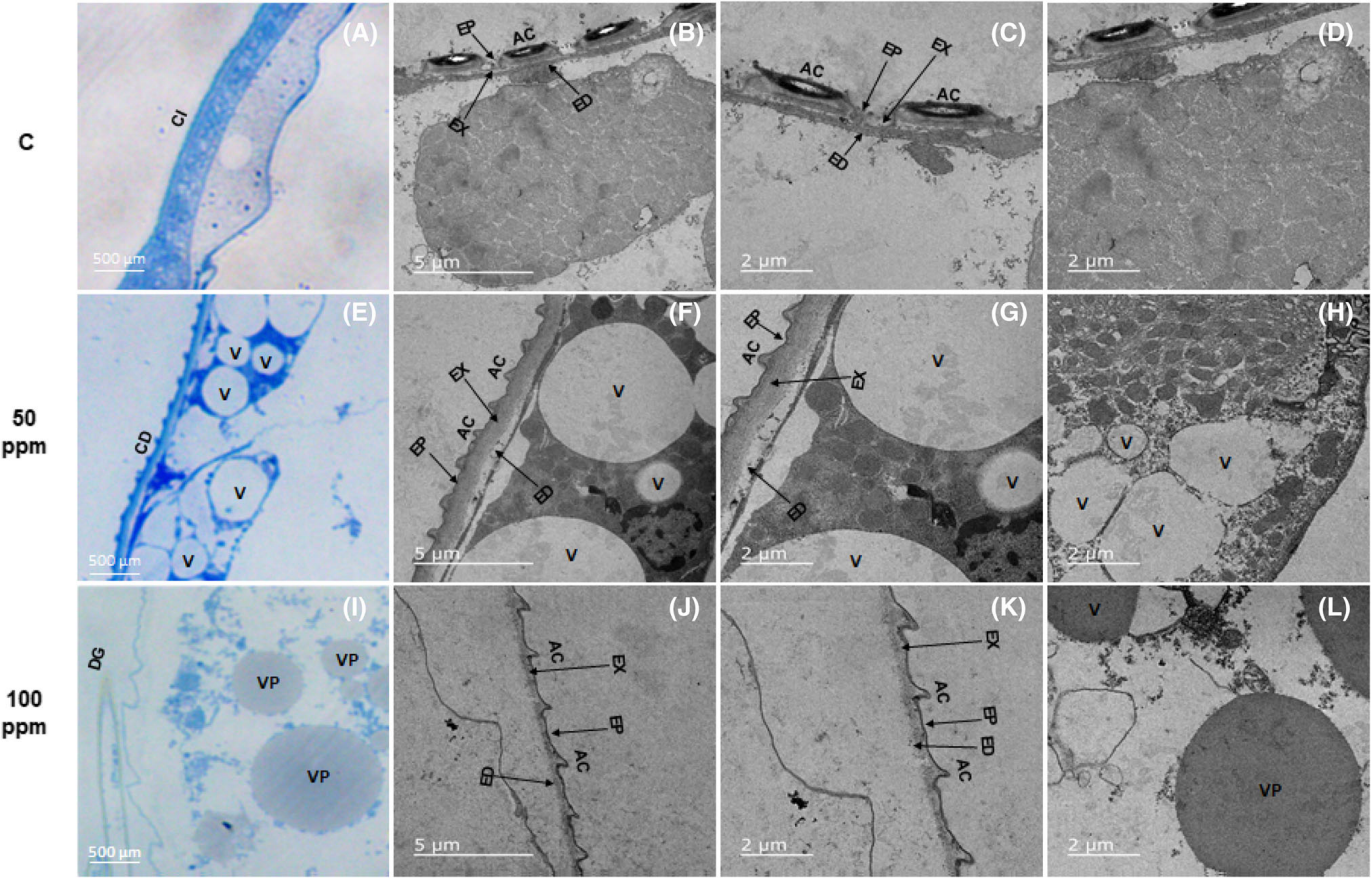
Optical microscopy (40×) and transmission electron microscopy (TEM) of the midgut region of third‐instar *Ae. aegypti* larvae. Panels C–D: untreated control; panels E–H: larvae treated with 50 ppm of the isolated compound **1**; panels I–L: larvae treated with 100 ppm of the isolated compound **1**. Abbreviations: CI, intact cuticle; CD, damaged cuticle; DG, severe cuticular damage; AC, aculei; EP, epicuticle; EX, exocuticle; ED, endocuticle; V, vacuoles; VP, filled vacuoles.

Histological analyses of larvae exposed to 50 and 100 ppm of **1** revealed pronounced cuticular disruption and vacuole formation (Fig. 8). At 50 ppm, damage to the cuticle (CD) in the midgut region was evident when compared to the control (Fig. 8(B), (C) *versus* Fig. 8(F), (G)), particularly affecting the cuticular aculei (AC). Higher‐magnification images (Fig. 8(C)–(H)) showed extensive vacuolization (V) in digestive epithelial cells relative to controls (Fig. 8(D)), indicating substantial cellular disturbance. At 100 ppm, more severe damage (DG) was observed, affecting the aculei (AC), exocuticle (EX), and endocuticle (ED) (Fig. 8(I)–(L)). Large vacuoles, likely containing lipid material (VP – Fig. 8(I), (L)), were also detected, corroborating the findings of Scudeler and Santos,^24^ who described lipid droplet accumulation in the midgut epithelium of *Ceraeochrysa claveri* larvae exposed to *Azadirachta indica* oil.

Cuticular disruption and vacuolization have been consistently described as typical responses to botanical larvicides. Similar alterations were previously reported^25^, ^26^ in *Ae. aegypti* larvae exposed to escamocin from *Annona squamosa*. Da Silveira and collaborators,^4^ who observed cuticular ruptures in larvae treated with enzymes from Trichoderma asperellum. Likewise, cuticular detachment and thoracic‐abdominal perforations were documented in larvae treated with rotenoids from *Clitoria fairchildiana* seeds.^14^

**Figure 9:**
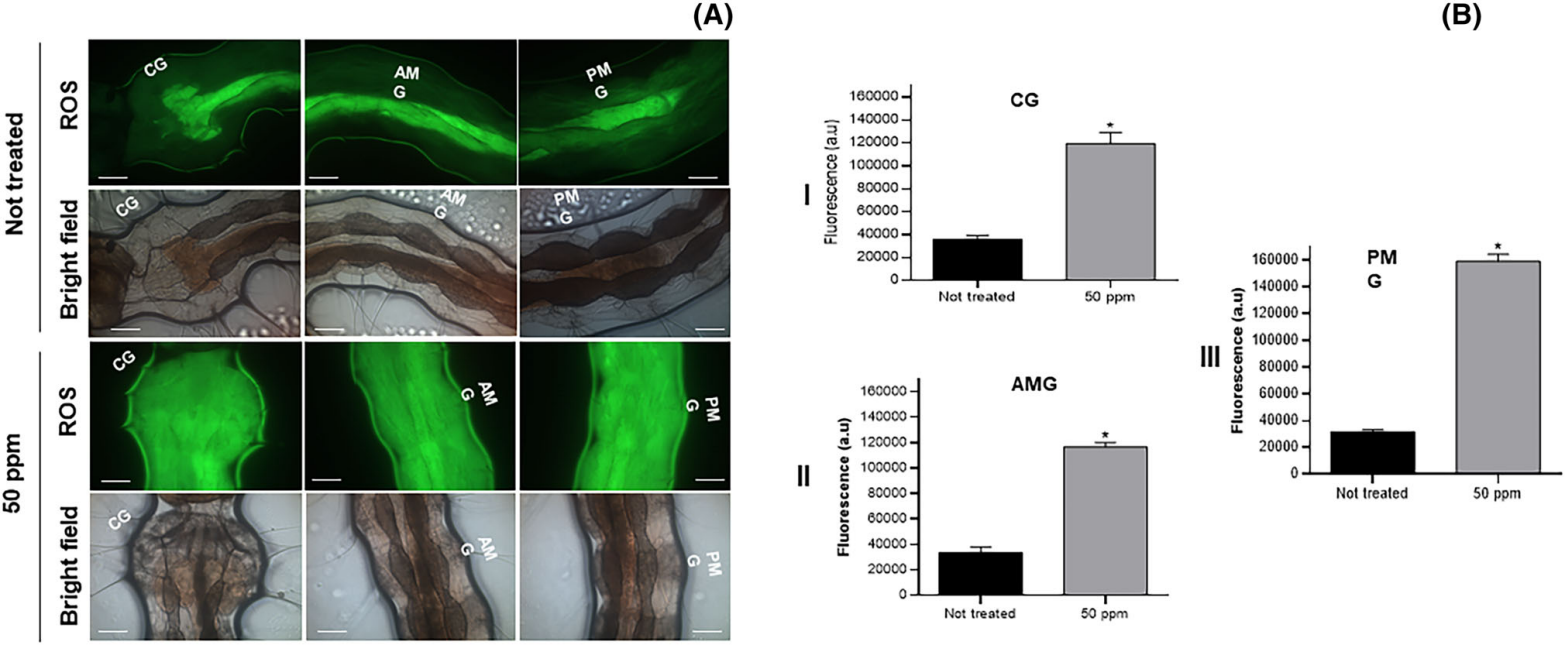
Oxidative stress in third instar larvae of *Ae. aegypti*. A‐Fluorescent labeling of ROS. The scale bar corresponds to 100 μm. B – Fluorescence quantification by Zen 2.3 software in CG – gastric cecum (I), AMG – anterior midgut (II) and PMG – posterior midgut (III) – GC of untreated larvae and in larvae treated with the isolated compound **1** (50 ppm). ROS – reactive oxygen species.

In addition, oxidative stress parameters were evaluated in third‐instar larvae exposed to 50 ppm of compound **1**. An increased production of reactive oxygen species (ROS) was detected throughout the larval body (Fig. 9(A)). In the gastric cecum (GC), anterior midgut (AMG), and posterior midgut (PMG), fluorescence intensity increased 3.3, 3.5, and 5‐fold, respectively, compared to the control group (Fig. 9(B)–(I), **II, III**). These findings are consistent with those of Bertonceli and collaborators,^14^ who demonstrated that oxidative stress in the larval midgut of *Ae. aegypti* is associated with structural damage and larval mortality.

## 4 CONCLUSIONS

This study reports the identification and structural characterization of a new protolimonoid, 17‐*epi*‐melianodiol (**1**), obtained from the dichloromethane fraction of the methanolic extract of *M. azedarach* fruits through bioassay‐guided fractionation. Compound **1** demonstrated relevant larvicidal activity against third‐instar *Ae. aegypti* larvae, with LC₅₀ and LC₉₀ values of 41.5 and 64.7 ppm, respectively.

Exposure of larvae to compound **1** resulted in pronounced morphological and ultrastructural alterations, including cuticular disruption and midgut vacuolization, accompanied by increased levels of reactive oxygen species (ROS) in larval tissues. These combined findings indicate the occurrence of oxidative imbalance associated with tissue damage and suggest that multiple physiological disturbances may contribute to larval mortality.

The structural novelty of 17‐*epi*‐melianodiol and the nocive effects reported in this study reinforce the potential of protolimonids as alternative agents for controlling *Ae. aegypti* and encourage further research into species of the genus Melia as sources of bioactive metabolites for vector management.

## CONFLICT OF INTEREST

The authors declare no conflict of interest.

## Supporting information


**Table S1.** ^1^H NMR (500 MHz), HSQC and HMBC data of **1**, in CDCl_3_. Shift values obtained in ppm principals).
**Fig. S1.** ^1^H NMR spectrum of **1** (500 MHz, CDCl_3_).
**Fig. S2.** ^13^C‐DEPTQ NMR spectrum of **1** (125 MHz, CDCl_3_).
**Fig. S3.** ^1^H—^1^H COSY spectrum of **1**.
**Fig. S4.** HSQC spectrum of **1**.
**Fig. S5.** HMBC spectrum of **1**.
**Fig. S6.** NOESY spectrum of **1**.
**Fig. S7. (A).** HRESI‐MS (Positive ionization) spectrum. **(B)** MS/MS spectrum of the m/z 511.3369 peak of **1**. **(C)** MS/MS spectrum of the m/z 999.6836 peak of **1**.
**Scheme S1.** Proposed fragmentation mechanisms of **1** (only peaks classified as principals).

## Data Availability

The data that support the findings of this study are available from the corresponding author upon reasonable request.
