# Supplementary material for: 17‐ epi ‐Melianodiol, a new protolimonoid from Melia azedarach fruits, exhibits larvicidal activity and is associated with oxidative imbalance and midgut epithelial damage in Aedes aegypti larvae
Source: Pest Manag Sci. 2026 May 27;82(9):9240–8. doi: 10.1002/ps.70968 (PMC13453259; doi:10.1002/ps.70968)
Supplement: Supplementary file 1 [file PS-82-9240-s001.docx]

# [Supporting Information](https://pubs.acs.org/doi/suppl/10.1021/acs.jnatprod.0c01062)

**17-*epi*-Melianodiol, a new protolimonoid from *Melia azedarach* fruits, exhibits larvicidal activity and is associated with oxidative imbalance and midgut epithelial damage in *Aedes aegypti* larvae**

*Kethleen Duarte Crespo Soares^1^*, Thalya Soares Ribeiro Nogueira^1^, Maria Aparecida Aride Bertonceli^2^, Renata Rodrigues da Silva Robaina^1^, Leticia Oliveira da Rocha^3^, Francisco José Alves Lemos^2^, Kátia Valevski Sales Fernandes^2^, Raimundo Braz-Filho^1,3^ and Ivo José Curcino Vieira^1^.*

^1^ Universidade Estadual do Norte Fluminense Darcy Ribeiro, Setor de Química dos Produtos Naturais, Laboratório de Ciências Químicas, Avenida Alberto Lamego 2000, CEP 28013-602, Campos dos Goytacazes-RJ, Brasil.

^2^ Universidade Estadual do Norte Fluminense Darcy Ribeiro, Laboratório de Química e Função de Proteínas e Peptídeos, Centro de Biociências e Biotecnologia, Avenida Alberto Lamego 2000, CEP 28013-602, Campos dos Goytacazes-RJ, Brasil

^3^ Universidade Estadual do Norte Fluminense Darcy Ribeiro, Laboratório de Biologia Celular e Tecidual, Centro de Biociências e Biotecnologia, Avenida Alberto Lamego 2000, CEP 28013-602, Campos dos Goytacazes-RJ, Brasil.

^4^ Departamento de Química, Instituto de Química, Universidade Federal Rural do Rio de Janeiro, CEP 20000-000, Seropédica-RJ, Brasil.

*** Corresponding author at:** K. D. C. Soares, Universidade Estadual do Norte Fluminense Darcy Ribeiro, Setor de Química dos Produtos Naturais, Laboratório de Ciências Químicas, Avenida Alberto Lamego 2000, CEP 28013-602, Campos dos Goytacazes-RJ, Brasil.

**e-mail address:** [duarte.cblab@gmail.com](mailto:duarte.cblab@gmail.com)

**CONTENTS OF SUPPORTING INFORMATION**

**LIST OF TABLE**

[**Table S1**. ^1^H NMR (500 MHz), HSQC and HMBC data of **1**, in CDCl_3_. Shift values obtained in ppm principals).	3](#_Toc139180790)

**LIST OF FIGURES**

[**Figure S1.** ^1^H NMR spectrum of **1** (500 MHz, CDCl_3_).	4](#_Toc139180413)

[**Figure S2.** ^13^C-DEPTQ NMR spectrum of **1** (125 MHz, CDCl_3_).	5](#_Toc139180414)

[**Figure S3.** ^1^H-^1^H COSY spectrum of **1**.	6](#_Toc139180415)

[**Figure S4.** HSQC spectrum of **1**.	7](#_Toc139180416)

[**Figure S5.** HMBC spectrum of **1**.	8](#_Toc139180417)

[**Figure S6.** NOESY spectrum of **1**.	9](#_Toc139180418)

[**Figure S7.** (**A)** HRESI-MS (Positive ionization) spectrum. (**B)** MS/MS spectrum of the m/z 511.3369 peak of **1**. (**C)** MS/MS spectrum of the m/z 999.6836 peak of **1**	10](#_Toc139180419)

**LIST OF SCHEMES**

[**Scheme S1**. Proposed fragmentation mechanisms of **1** (only peaks classified as principals).	11](#_Toc139180790)

|  | **Compound 1** | | | |
| --- | --- | --- | --- | --- |
|  | **HSQC** | | **HMBC** | |
|  | ***δ*_c_** | ***δ*_H_** | **^2^*J*_CH_** | **^3^*J*_CH_** |
| **C** |  |  |  |  |
| 3 | 216.49 | - | H-2a | H-1a; 3H-28; 3H-29 |
| 4 | 47.86 | - | 3H-28;  3H-29 |  |
| 8 | 146.67 | - |  | 2H-6; 3H-30 |
| 10 | 35.11 | - |  | 3H-19 |
| 13 | 43.55 | - | 3H-18 | 3H-30 |
| 14 | 50.09 | - | 3H-30 | 3h-18 |
| 25 | 73.37 | - | 3H-26; 3H-27- | - |
| **CH** |  |  |  |  |
| 5 | 52.47 | 1.74 (t, 7.6) |  | 3H-19; 3H-28; 3H-29 |
| 7 | 118.18 | 5.34 (m) | 2H-6 |  |
| 9 | 48.40 | 2.31 (m) |  | 3H-19 |
| 17 | 45.36 | 2.05 | - | 3H-18- |
| 20 | 46.28 | 2.01 |  |  |
| 21 | 97.69 | 5.22. (d, 2.2) |  |  |
| 23 | 78.64 | 4.49 (t, 7.8) |  |  |
| 24 | 75.32 | 3.19 (s) |  | 3H-26; 3H-27 |
| **CH_2_** |  |  |  |  |
| 1 | 38.52 | 2.01, 1.49 | H-2a | 3H-19 |
| 2 | 34.24 | 2.77 (dd, 14.6, 5.5)  2.26 dt, 14.6, 3.5) |  |  |
| 6 | 24.77 | 2.15, 2.05 |  |  |
| 11 | 17.77 | 1.60 (m) |  | 3H-18 |
| 12 | 31.50 | 1.75, 1.45 |  |  |
| 15 | 34.88 | 1.55 |  | 3H-30 |
| 16 | 27.24 | 2.0-1.90 |  |  |
| 22 | 30.32 | 2.02, 1.92 |  |  |
| **CH_3_** |  |  |  |  |
| 18 | 23.22 | 0.86 (s) |  |  |
| 19 | 12.73 | 1.03 (s) |  | H-5 |
| - | - | - | - | - |
| 26 | 26.61 | 1.30 (s) |  | 3H-27 |
| 27 | 26.69 | 1.28 (s) |  | 3H-26 |
| 28 | 24.53 | 1.05 (s) |  | H-5; 3H-29 |
| 29 | 21.57 | 1.13 (s) |  | H-5; 3H-28 |
| 30 | 27.46 | 1.06 (s) |  |  |

**Table S1.** ^1^H NMR (500 MHz), HSQC and HMBC data of compound **1**, in CDCl_3_. Shift values obtained in ppm.

**Figure S1.** ^1^H NMR spectrum of **1** (500 MHz, CDCl_3_).

**Figure S2.** ^13^C-DEPTQ NMR spectrum of **1** (125 MHz, CDCl_3_).

**Figure S3.** ^1^H-^1^H COSY spectrum of **1**.

**Figure S4.** HSQC spectrum of **1**.

**Figure S5.** HMBC spectrum of **1**.

**Figure S6.** NOESY spectrum of **1**.

**Figure S7.** (**A)** HRESI-MS (Positive ionization) spectrum. (**B)** MS/MS spectrum of the *m/z* 511.3328 peak of **1**. (**C)** MS/MS spectrum of the *m/z* 999.6757 peak of **1.**

**Scheme S1**. Proposed fragmentation mechanisms of **1** (only peaks classified as principals)
